# Laboratory Investigation of Shear Behavior of High-Density Polyethylene Geomembrane Interfaces

**DOI:** 10.3390/polym10070734

**Published:** 2018-07-04

**Authors:** Wei-Jun Cen, Hui Wang, Ying-Jie Sun

**Affiliations:** College of Water Conservancy and Hydropower Engineering, Hohai University, Nanjing 210098, China; whhhuwh@163.com (H.W.); sunyjhhu@163.com (Y.-J.S.)

**Keywords:** geomembrane interfaces, peak shear strength, residual shear strength, large-scale composite shear apparatus, direct shear test

## Abstract

As a product of polymeric materials, geomembranes (GMs) are widely used in engineered systems as impervious barriers due to their low permeability. In this study, a large-scale composite shear test apparatus was developed to investigate the shear behaviors of various GM interfaces. A series of direct shear tests were conducted on GM–soil, GM–geotextile, and GM–concrete interfaces. Two types of high-density polyethylene (HDPE) GMs, a smooth GM and a textured GM, were used to evaluate the effect of GM-texturing on the shear properties of these interfaces. Based on the experimental data, the friction angles and adhesions of GM interfaces were calculated using the Mohr–Coulomb criterion. Test results describing the behavior of GM–soil and GM–geotextile interfaces from the current study were then compared with results from previous studies. The test results are shown to verify the reliability of the new large-scale composite shear apparatus. In addition, this paper presents preliminary experimental results of the GM–concrete interface shear tests.

## 1. Introduction 

As a product of polymeric materials, geomembranes (GMs) are widely used in the environmental, geotechnical, hydraulic, and transportation sectors as barrier layers with low permeability, e.g., landfill basal liners or capping [1], tailings ponds, or leaching ponds in mineral and ore processing, dams or dykes, reservoirs, canal construction, tunnel construction, and large-area contiguous liners in road construction [2]. These GM barriers can effectively minimize the penetration of liquids into the engineered systems. In practical design, GMs are generally combined with soils in the impervious liners or at the boundary of a drainage layer. To protect GMs from puncture and tear caused by angular particles, geotextiles (GTs) are commonly used in conjunction with GMs to form composite GM–GT lining systems [3]. In addition, GMs are increasingly being used in the remediation of existing concrete dams suffering from leakage damage, and in rockfill dams as the impervious barrier on the upstream dam surface. For the latter use case, ordinary concrete slabs are commonly placed over the GMs to form a protective layer, or no-fines concrete can be used as an underlying drainage layer beneath the GM barrier. For the GM applications mentioned above, the interfaces between GMs and soils, GTs, or concrete require substantial attention during design. These GM interfaces may exhibit low shear resistance, and as such could become a potential source of failure. Therefore, the accurate assessment of the shear strength of GM interfaces is necessary, and laboratory tests can provide an effective means to evaluate the shear behavior of GM interfaces. 

Currently, the methods typically used to investigate the shear behavior of GM interfaces include the inclined shear test, torsional ring shear test, and direct shear test. The inclined shear test can accurately reproduce the actual conditions in the lining system, where the normal stresses are generally low. Izgin and Wasti [4] measured the shear strength parameters of the GM–sand interface using the inclined shear test, considering the effect of sand particle size on the determined interface friction angles. Similar tests were conducted by Wasti and Özdüzgün [5] to obtain the shear strength parameters of the GM–GT interface. However, high normal stresses are not conveniently applied in the inclined board test because a tall sliding block is required, and a significant overturning moment is frequently produced. Thus, the torsional ring shear test and direct shear test have served in the past as suitable alternatives when evaluating high normal stress cases.

Owing to the advantages of the torsional ring shear test apparatus, such as unlimited continuous shear displacement, constant cross-sectional area during shearing, and convenient data acquisition, multiple torsional ring shear tests have been reported in previous studies of the shear properties of the GM interface. Stark et al. [6] described the results of torsional ring shear tests on GM–GT interfaces considering the effect of GT fiber type, fabric style, and mass per unit area. Jones and Dixon [7] conducted a series of ring shear tests to investigate the factors regulating the shear strength of GM–GT interfaces. Eid [8] performed torsional ring shear tests on a GM–geosynthetic clay liner (GCL) interface to determine the relationship between the shear strength and magnitude of normal stress. The torsional ring shear test is often an effective method for evaluating the shear behavior of GM interfaces; however, a shear displacement of 40–60 cm is typically required before the residual interface shear strength can be mobilized [9]. This can often necessitate substantial time for the completion of the shear test. However, the classic direct shear apparatus is widely considered to provide a reasonable estimate of peak strength, and the peak strength is mobilized at a comparatively more marginal shear displacement than is required for the torsional ring shear test [10]. 

Over the past few decades, many direct shear tests have been conducted on GM interfaces [11,12,13,14,15,16,17,18,19,20,21,22,23,24,25,26,27,28,29,30], with various factors being considered during these tests. In the studies mentioned above, different types of standard-sized or large-scale direct shear apparatuses were employed to simulate some specific interface types, such as between a GM and soil, GT, or GCL. The differences between these apparatuses affect the ability to accurately compare tests and often increase the test cost. Thus, the development of a large-scale composite shear apparatus can provide great convenience and save much cost for accurately and consistently simulating the shear behaviors of various GM interfaces.

In this study, a large-scale composite shear apparatus with alternative upper shear boxes was developed to conduct direct shear tests on several types of GM interfaces, including a GM–fine sand (GM–FS) interface, GM–sandy gravel (GM–SG) interface, GM–geotextile (GM–GT) interface, GM–ordinary concrete (GM–OC) interface, and GM–no-fines concrete (GM–NFC) interface, all in accordance with the requirements of ASTM D5321/D5321M-14 [31]. Both smooth and textured high density polyethylene (HDPE) GMs were used to investigate the influence of GM texturing on the shear strengths of the different GM interfaces tested. The shear stress versus shear displacement curves were automatically captured during the tests, and the friction angle and adhesion of each interface were calculated using the Mohr-Coulomb criterion. The test results of GM–soil and GM–GT interfaces were compared with the results of previous studies to verify the accuracy of the proposed apparatus, and to present a summary of the shear properties of the GM interfaces evaluated. Further, the shear strength parameters of GM–concrete interfaces were provided as the results of a preliminary investigation.

## 2. Test Apparatus and Scheme

**Figure 1:**
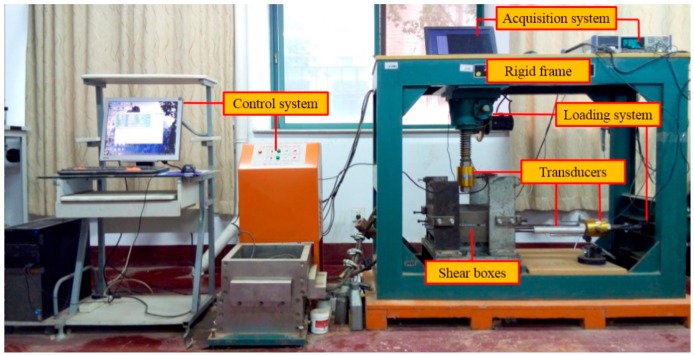
Photograph of the large-scale composite direct shear apparatus.

**Figure 2:**
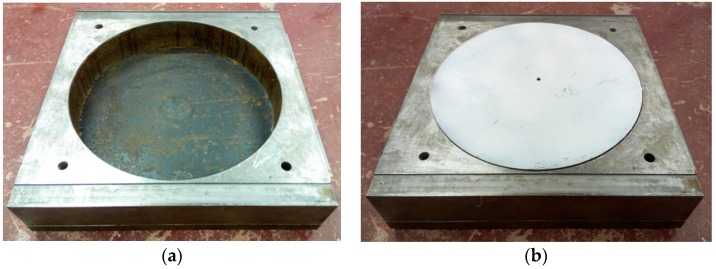
Photographs of lower shear box: (**a**) With cylindrical hole; and (**b**) With cylindrical block.

**Figure 3:**
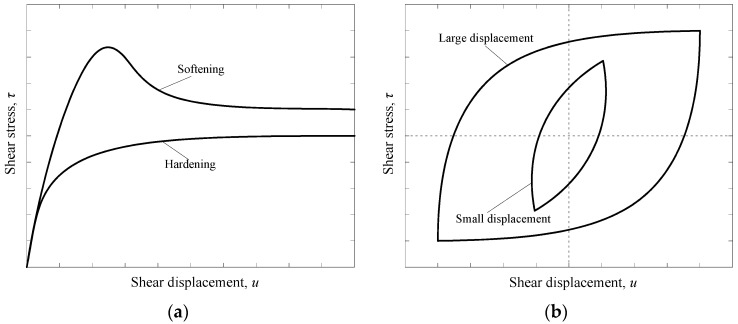
Typical shear test curves for: (**a**) Monotonic shear behavior; and (**b**) Cyclic shear behavior.

A large-scale displacement-controlled composite shear test apparatus was developed to simulate the shear behaviors of various GM interfaces. Both monotonic and cyclic shear tests can be conducted using this apparatus. The apparatus consists of loading and control device, shear boxes, and a data acquisition system (Figure 1). A rigid frame of size 1400 mm × 500 mm × 1100 mm is equipped to provide the reflexive normal and shear forces. Three alternative upper shear boxes were designed for the shear tests of the different GM interfaces. A few details of this apparatus are described below:

- Upper shear box: ① For the GM–soil interface shear tests, a 360 mm × 360 mm × 100 mm upper square box with an inner cylindrical hole of diameter 300 mm was employed. The cylindrical loading area was chosen to avoid stress concentration. The soils were poured into cylindrical hole of the upper box in several layers and compacted using a hammer to attain the design density. ② For the GM–GT interface shear tests, the geotextile sample was fixed on a rigid, trapezoidal block that served as the upper box, and was then placed on the lower box. The upper box had smaller dimensions than the lower box to prevent displacement-induced loss of the area of the GM–GT interface during measurement. ③ For the GM–concrete interface shear tests, the concrete blocks were prepared with dimensions of 300 mm × 300 mm × 100 mm and were placed directly on the lower box during the shear tests. All upper shear boxes were fixed while the lower shear box moved horizontally. All upper shear boxes were fixed in space in horizontal direction, but it could freely move vertically during shearing.
- Lower shear box: The lower shear box was made of a 360 mm × 360 mm × 80 mm rigid block with an inner cylindrical hole of diameter 300 mm. A rigid cylindrical block was inserted in this cylindrical hole to form a smooth horizontal plane (Figure 2) when the GM interface shear tests are conducted. The GM sample was cut into a rectangle of size 480 mm × 300 mm. The rectangle sample was then glued onto the lower box and laterally clamped using four bolts and two steel blocks to prevent the sample from sliding. During the whole shearing process, there is no area loss for different GM interfaces. Further, when the rigid cylindrical block is removed, it could also be used for soil shearing tests with a corresponding upper shear box.
- Loading system: The loading system was comprised of a vertical actuator and a horizontal actuator. Vertical pressure was applied to the upper shear box through a pressure rod within a range of 0 kN to 100 kN. The shear force was horizontally applied to the lower shear box through a pull-rod at a displacement-controlled shear rate between 0.01 mm/min and 5.00 mm/min. The pull-rod could also act as a push-rod when the shear direction was reversed in cyclic shear tests. The precision error of the loading system was less than 1%.
- Acquisition system: Pressure and displacement transducers were employed for the automatic acquisition of the normal pressure, shear force, and shear displacement. The experimental data were recorded using a data logger. A personal computer was used to control the data logger and to store and manipulate the recorded information. The test curves of normal stress versus shear displacement were plotted automatically. Figure 3 shows the typical test curves for both monotonic and cyclic shear tests.

In this study, a series of monotonic shear tests on a variety of GM interfaces (GM–FS, GM–SG, GM–GT, GM–OC, and GM–NFC) were conducted under normal stresses of 50, 100, 150, and 200 kPa with virgin samples used for each vertical pressure. The vertical pressure was held for several minutes prior to the commencement of the shearing test until it stabilized. A shear rate of 1 mm/min was set for all shear tests. The shear displacements and shear forces were recorded at 2 s intervals until the shear force resisted by the interface showed no further significant change.

## 3. Test Materials

**Figure 4:**
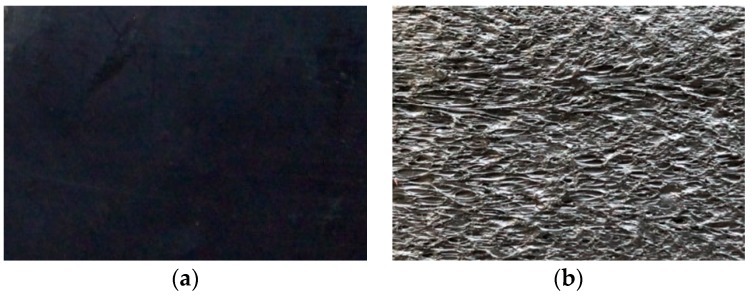
Photographs of geomembranes (GMs): (**a**) Smooth GM (GM(S)); and (**b**) textured GM (GM(T)).

**Figure 5:**
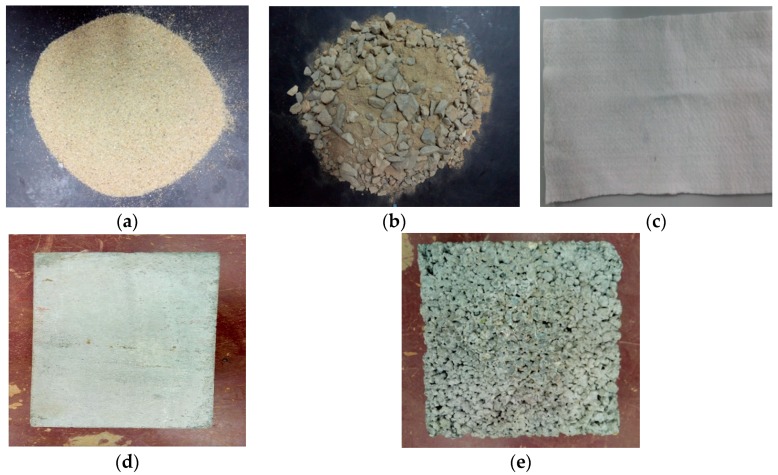
Photographs of materials used: (**a**) Fine sand (FS); (**b**) Sandy gravel (SG); (**c**) Geotextile (GT); (**d**) Ordinary concrete (OC); and (**e**) No-fines concrete (NFC).

Smooth and textured HDPE GMs with a nominal thickness of 2 mm and a density of 0.94 g/cm^3^ (Figure 4) were used in the tests. The symbols GM(S) and GM(T) in this paper signify smooth and textured GMs, respectively. Figure 5 presents the materials in contact with the GM in the shear tests. The GTs used in the tests have a mass per area of 300 g/m^2^. The physical properties of the soils and concretes used are listed in Table 1 and Table 2.

## 4. Test Results

### 4.1. GM–Soil Interface

**Figure 6:**
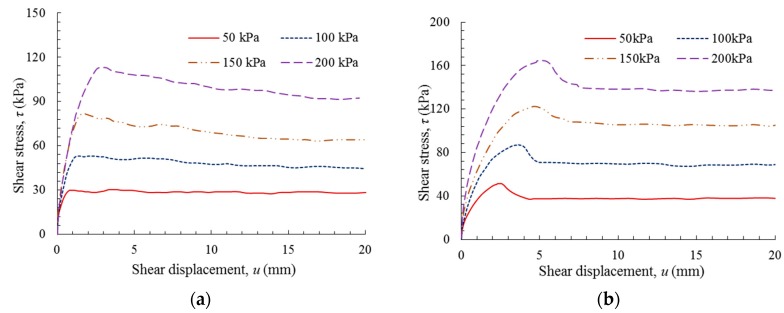
Shear stress versus shear displacement: (**a**) GM(S)–FS interface; and (**b**) GM(T)–FS interface.

Figure 6 shows the plots of shear stress versus shear displacement for the GM(S/T)–FS interfaces. Owing to the influence of GM texturing, the shear strength curves of these two interfaces exhibited dissimilar shapes. After the GM(S)–FS interface attained its peak shear stress, the shear stress gradually decreased to a stable value, while the peak shear stress of the GM(T)–FS interface rapidly dropped to a residual value where it remained stable under further shear displacement. It can be observed that the GM(T)–FS interface exhibited higher peak and residual shear stresses than the GM(S)–FS interface.

**Figure 7:**
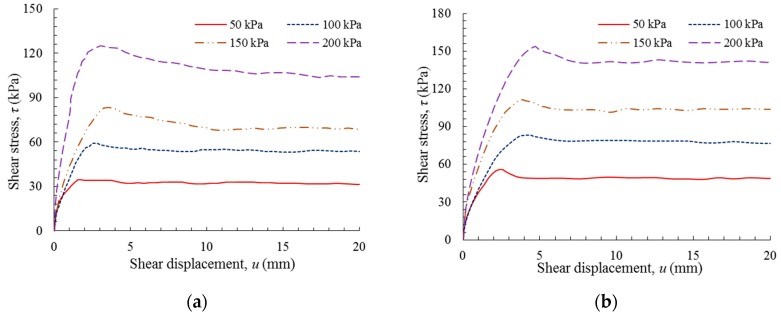
Shear stress versus shear displacement: (**a**) GM(S)–SG interface; and (**b**) GM(T)–SG interface.

Figure 7 shows the shear behavior of the GM(S/T)–SG interface. Similar to the GM–FS interface, the GM texturing also exerted an apparent influence on the shear stress versus shear displacement curves. Compared to the GM(S)–SG interface, higher peak and residual shear stresses were obtained for the GM(T)–SG interface under a similar applied normal stress. The reduction in the peak shear stress was more significant for the GM–SG interface than for the GM–FS interface, as observed by comparing Figure 6 and Figure 7. The differences in the experimental data from the GM(S/T)–FS and GM(S/T)–SG interface tests also revealed that the interface shear behaviors were influenced by both the particle size and gradation of the soils. For soils with large and angular particles, e.g., SG, they can presented relatively high shear strength of GM(S/T)-soil interface due to the interlock mechanism between particles and geomembranes.

### 4.2. GM–GT Interface

**Figure 8:**
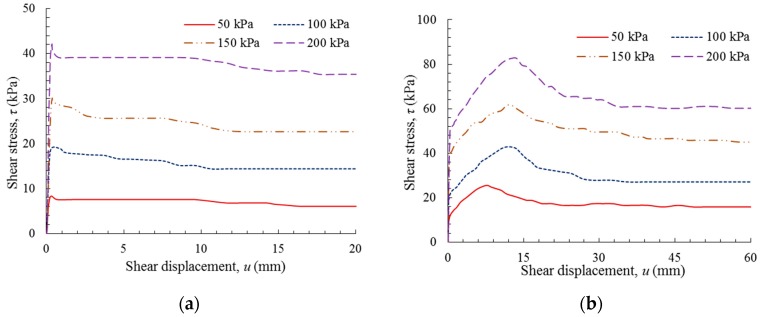
Shear stress versus shear displacement: (**a**) GM(S)–GT interface; and (**b**) GM(T)–GT interface.

Figure 8 shows the shear behavior of the GM(S/T)–GT interface. For the GM(S)–GT interface, shown in Figure 8a, there was an initial sharp increase in shear stress as soon as the shear displacement began, followed by a marginal loss of peak shear stress with further shear displacement. The peak shear stress of this interface typically occurred at a shear displacement of less than 0.4 mm, with the shear stress decreasing by 20–30% to the residual stress within a shear displacement of approximately 10–15 mm.

Figure 8b shows the relationship between shear stress and shear displacement for the GM(T)–GT interface. In contrast with the GM(S)–GT interface, the shear stresses increased to peak values through two distinct phases. The peak shear strength of the GM(T) interface typically occurred at a displacement between 6 mm and 13 mm, which was a significantly larger displacement than that exhibited by the GM(S) interface at peak shear strength, depending on the magnitude of the applied normal stress (50–200 kPa). Compared to the GM(S)–GT interface, the GM(T)–GT interface exhibited a more evident softening behavior, with a 25–40% reduction in peak shear stress within a residual shear displacement of approximately 20–50 mm.

### 4.3. GM–Concrete Interface

**Figure 9:**
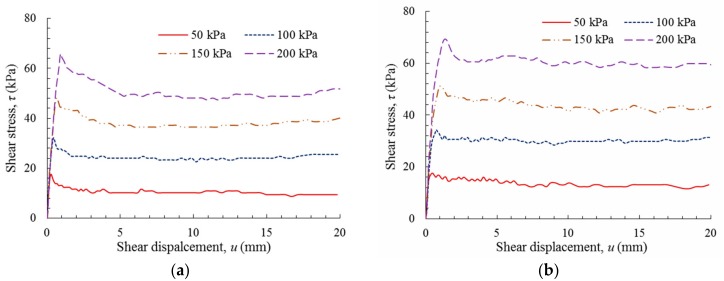
Shear stress versus shear displacement: (**a**) GM(S)–OC interface; and (**b**) GM(T)–OC interface.

Figure 9 shows the shear stress versus shear displacement curves for the GM(S/T)–OC interfaces. The shear stresses of these two interfaces increased sharply before attaining their peaks, after which softening behavior occurred. The peak shear stresses of the GM(S)–OC interface were typically 1–5 kPa lower than that of the GM(T)–OC interface, depending on the normal stress applied (50–200 kPa). The shear displacements corresponding to the peak shear stresses of the GM(S)–OC interface were approximately 0.3 mm smaller than those of the GM(T)–OC interface. The reductions of the peak shear stresses of the GM(S)–OC interface were approximately identical to those of the GM(T)–OC interface. In all, the GM-texturing exhibited negligible influence on the shear behavior of the GM–OC interface.

**Figure 10:**
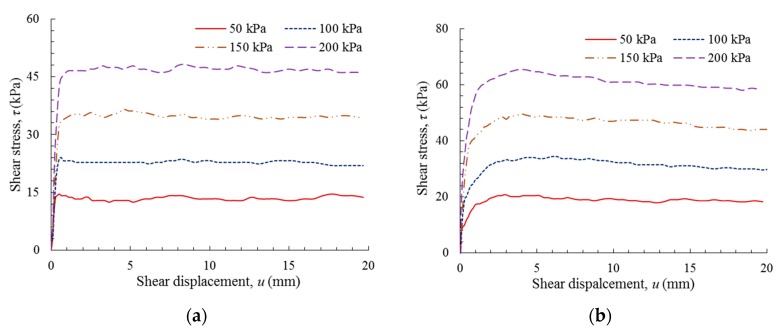
Shear stress versus shear displacement: (**a**) GM(S)–NFC interface; and (**b**) GM(T)–NFC interface.

Figure 10 shows the shear behavior of the GM(S/T)–NFC interface. It was observed that the shear behavior of the GM(S)–NFC interface was distinct from that of the GM(T)–NFC interface. While both interfaces exhibited an initial sharp increase, for the GM(S)–NFC interface, the peak shear stresses were typically attained at a shear displacement below 1.2 mm, while for the GM(T)–NFC interface, the shear stresses continued to gradually increase to peak values at a shear displacement in the range of 2.5–4 mm. The peak shear stresses corresponding to normal stresses of 50, 100, 150, and 200 kPa of the GM(S)–NFC interface are 14.57, 24.10, 35.31, and 46.52 kPa, respectively, while the peak shear stresses of the GM(T)–NFC interface were 20.45, 34.30, 49.26, and 65.68 kPa, respectively. This indicates that the GM texturing increased the peak shear stress values and the corresponding shear displacements for a GM–NFC interface. Subsequent to the peak, the shear stress values of these two interfaces fundamentally remained stable, and softening behaviors were not evident. In effect, the interface resistances remained approximately constant even at a large shear displacement.

**Figure 11:**
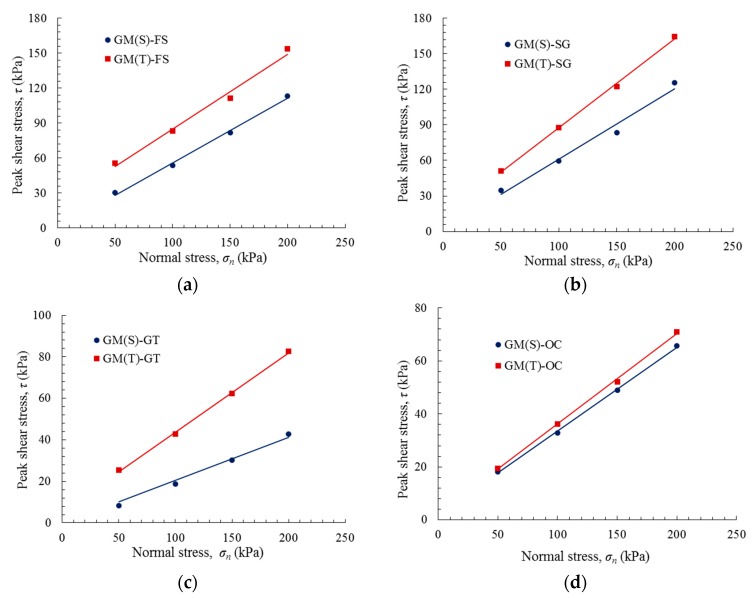

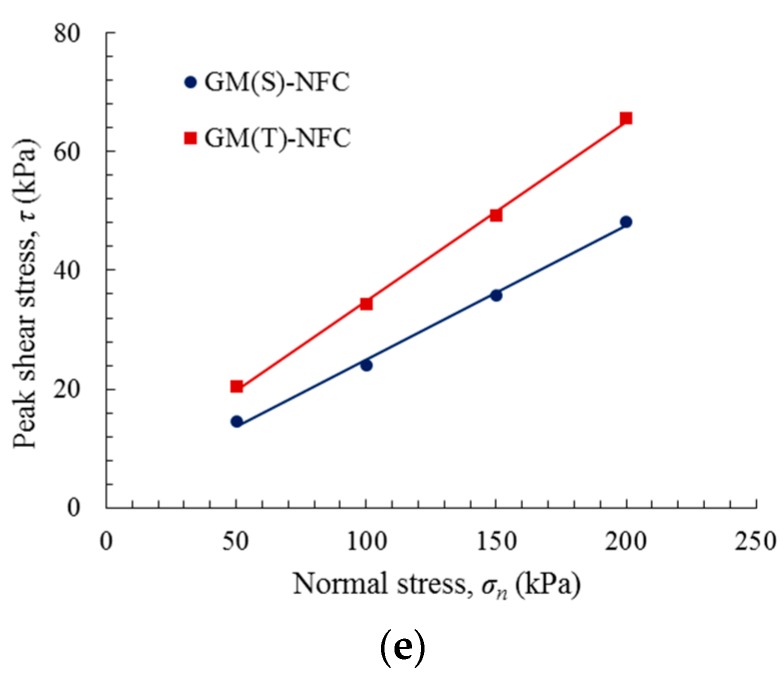
Peak shear stress versus normal stress: (**a**) GM–FS interface; (**b**) GM–SG interface; (**c**) GM–GT interface; (**d**) GM–OC interface; and (**e**) GM–NFC interface.

**Figure 12:**
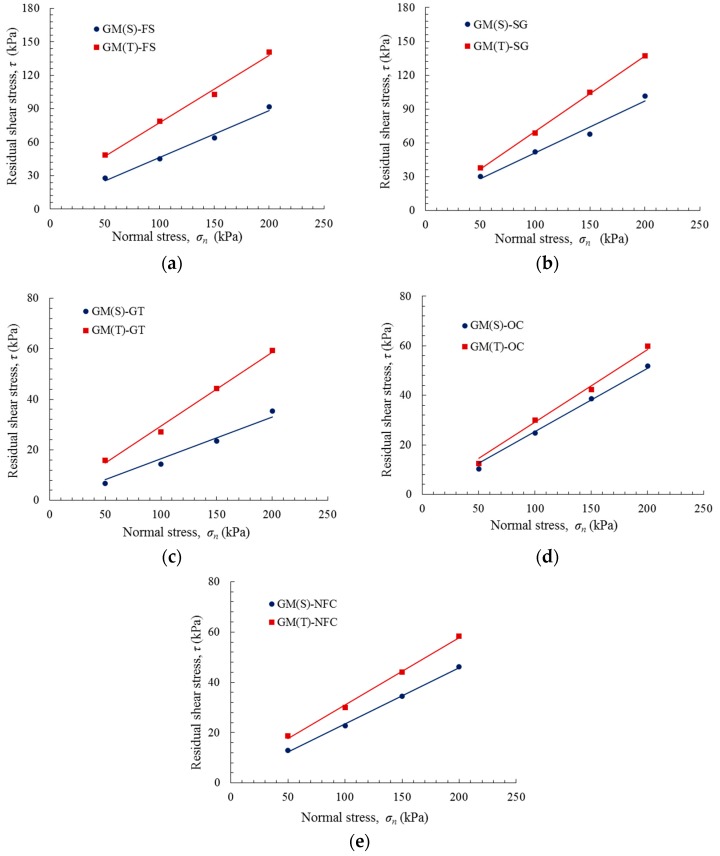
Residual shear stress versus normal stress: (**a**) GM–FS interface; (**b**) GM–SG interface; (**c**) GM–GT interface; (**d**) GM–OC interface; and (**e**) GM–NFC interface.

The peak and residual shear strength envelopes of the various GM interfaces are plotted in Figure 11 and Figure 12. These envelopes can be expressed as a function of the normal stress using the Mohr–Coulomb criterion:

(1) $$\tau =c+\sigma_{n}\tan\delta$$

where τ is the peak or residual shear stress, c is the peak or residual adhesion, $\sigma_{n}$ is the normal stress, and δ is the peak or residual friction angle.

Table 3 summarizes the shear strength parameters for the peak and residual envelopes of several GM interfaces using a regression analysis of the experimental data presented in Figure 11 and Figure 12. The correlation coefficients are all between 0.96 and 1.00. It was observed that the friction angles of the GM(T) interfaces are typically higher than those of the GM(S) interfaces, and that the residual friction angle of each interface is 2–6° lower than the peak friction angle. The GM(T) interfaces exhibited higher peak adhesions.

## 5. Comparison and Discussion

### 5.1. GM–Soil Interface

In this study, the friction angles of the GM–FS/SG interfaces are in a range of typical values published by Izgin and Wasti [4], Frost et al. [22], and Stark and Santoyo [32]. The use of textured GM in the GM(T)-soil tests obviously increases the friction angles by 12–15% compared with the GM(S)–soil interface. Additionally, the friction angles of the GM(S)–SG interface are approximately 2°–4° higher than those of the GM(S)–FS interface. It is therefore noted that the shear resistances of GM–soil interfaces are heavily influenced by GM texturing, as well as by the gradation and particle size of the soil. These are in accord with the general conclusion from previous studies summarized in Table 4. Undoubtedly, the comparison of test results is bound to reveal a few discrepancies between this paper and previous research as previous studies used soil with different physical properties.

### 5.2. GM–GT Interface

Table 5 presents a comparison of the present experimental results produced using the proposed large-scale composite shear test apparatus with the findings of the limited published work detailing the shear testing of GM(S/T)–GT interface. The friction angles of the GM(T)–GT interfaces determined by the current study are markedly higher than those of the GM(S)–GT interfaces. Note that the peak friction angle (11.61°) of the GM(S)–GT interface in the present study is adequately consistent with the values obtained by Wasti and Özdüzgün [5] and Akpinar and Benson [34]. Owing to the variations in the shear apparatuses employed, the range of normal stresses applied, and the physical properties of the geosynthetics used, a few discrepancies between the present results and reported studies can be observed.

### 5.3. GM–Concrete Interface

Limited investigations have been conducted on the shear behaviors of GM–concrete interfaces. However, the stability assessment of the GM–concrete interface is also of importance in practical engineering, particularly in dam engineering. Therefore, in place of comparison with extant research data, this paper presents the preliminary experimental results of the GM–concrete interface shear tests. Similar to the GM–soil and GM–GT interfaces, the friction angles of the GM(S)–concrete interface are typically lower than those of the GM(T)–concrete interface. Additionally, though the coarse aggregate content of the NFC is higher than that of the OC, the friction angles of the GM–NFC interfaces are 2°–5° lower than those of the GM–OC interfaces. This difference may be attributed to the fact that the effective contact per unit area of the GM–NFC interface is smaller than that of the GM–OC interface; the angularity of the aggregates is not the main factor governing the interface shear behavior.

## 6. Conclusions

In this study, a large-scale composite shear apparatus was developed and a series of monotonic shear tests were conducted on various types of GM interfaces. Two types of HDPE GMs, a smooth GM and a textured GM, were used to investigate the effect of GM texturing on the shear properties of GM interfaces. The friction angles and adhesions of various GM interfaces were compared to those determined in previous studies. The test results demonstrate that the developed shear apparatus can be effectively employed to investigate the shear behaviors of various types of GM interfaces. Based on the test results, the following conclusions can be drawn:

(1) The shear strength curves of GM–soil interfaces are clearly influenced by GM texturing. Compared to GM(S)–soil interfaces, higher peak shear stresses and corresponding shear displacements were observed for the GM(T)–soil interfaces. In general, the friction angles of the GM(T)–soil interfaces are 12–15% higher than those of GM(S)–soil interfaces. The friction angles of the GM–FS interface are typically lower than those of the GM–SG interface, owing to the effect of the gradation and particle size of these two types of soil. When compared to the results of previous studies, the strength parameters of the present study are in the range of typically observed values.
(2) The shear behavior of the GM–GT interface is also affected by GM texturing. The experimental curves present an apparent difference in behavior between the GM(S)–GT and GM(T)–GT interfaces. The GM(T)–GT interface exhibits a more evident softening behavior, with a higher reduction in the peak shear stress than the GM(S)–GT interface. The friction angles of the GM(T)–GT interface are 6°–8° higher than those of the GM(S)–GT interface. The peak friction angle of the GM–GT interface in this study is approximately equal to the values in some previously reported studies.
(3) The peak friction angle and adhesion of the GM(S)–OC interface are 17.57° and 1.83 kPa, respectively, and those of the GM(T)–OC interface are 18.81° and 2.1 kPa, respectively. Therefore, it can be stated that GM texturing exerts a negligible influence on the peak shear behavior of the GM–OC interface. By contrast, GM texturing increases the peak shear strength of the GM–NFC interface. The post-peak shear resistances of the GM–NFC interfaces remain approximately constant, notwithstanding the large shear displacement. Additionally, the friction angles of the GM–NFC interfaces are 2°–5° lower than those of the GM–OC interfaces, which may be attributed to the fact that the effective contact per unit area of the GM–NFC interface is smaller than that of the GM–OC interface.

In summary, the large-scale composite shear apparatus used in this study provided reasonable test results of different GM interfaces. The comparison between present and previous studies for GM-soil and GM-GT interfaces can give technical guidance for engineer design and construction. Additionally, the preliminary test results of GM-concrete interface make up for the lack of data about this type of interface, and further investigation on shear properties of GM-concrete interface still needs to be conducted by laboratory tests or theoretic analysis.

## Figures and Tables

**Table 1:** Physical properties of the test soils.

Soils	Density (g/cm^3^)	*d*_10_ (mm)	*d*_30_ (mm)	*d*_60_ (mm)	Coefficients of Uniformity *C_u_*	Coefficients of Curvature *C_c_*
Fine sand (FS)	1.77	0.16	0.34	0.65	4.06	1.11
Sandy gravel (SG)	1.96	0.16	1.42	7.50	46.86	1.68

**Table 2:** Physical properties of the test concretes.

Concretes	Size Range of Aggregate (mm)	Porosity (%)	Water–Cement Ratio (w/c)	Unit Weight (kg/m^3^)		
				Water	Cement	Aggregate
Ordinary concrete (OC)	5–10	-	0.5	190	404	1806
No-fines concrete (NFC)	5–20	20	0.3	113	378	1343

**Table 3:** Shear strength parameters of GM interfaces.

Interface	Peak Shear Strength			Residual Shear Strength		
	Friction Angle δ (°)	Adhesion c (kPa)	Correlation Coefficient *R^2^*	Friction Angle δ (°)	Adhesion c (kPa)	Correlation Coefficient *R^2^*
GM(S)–FS	28.96	0.50	0.9891	22.75	4.66	0.9857
GM(T)–FS	32.72	20.65	0.9875	31.14	17.25	0.9926
GM(S)–SG	30.62	1.86	0.9875	24.64	5.59	0.9769
GM(T)–SG	36.81	12.79	0.9983	33.81	3.35	0.9991
GM(S)–GT	11.61	0	0.9841	10.75	0	0.9684
GM(T)–GT	20.88	5.62	0.9981	16.37	0	0.9937
GM(S)–OC	17.57	1.83	0.9990	14.32	0	0.9919
GM(T)–OC	18.81	2.1	0.9985	16.29	0	0.9920
GM(S)–NFC	12.68	2.52	0.9965	12.54	1.26	0.9986
GM(T)–NFC	16.77	4.76	0.9985	14.94	4.39	0.9971

**Table 4:** Summary of GM(S/T)–soil interface shear strength parameters from previous studies.

Source	Normal Stress (kPa)	Interface	Peak shear Strength		Residual Shear Strength	
			Friction Angle δ (°)	Adhesion c (kPa)	Friction Angle δ (°)	Adhesion c (kPa)
Mitchell et al. [11]	158, 316, 479	GM(S)–concrete sand	18	-	-	-
		GM(S)–Ottawa sand	18	-	-	-
		GM(S)–Misa Schist sand	17	-	-	-
Izgin and Wasti [4]	5–50	GM(S)–Ottawa sand	22	2.76	-	-
		GM(T)–Ottawa sand	32	5.00	-	-
		GM(S)–Ottawa stone	31	4.25	-	-
		GM(T)–Ottawa stone	37	2.89	-	-
Bergado et al. [16]	150–400	GM(S)–compacted clay	10.5	-	-	-
Fleming et al. [17]	-	GM(S)–Silty sand	21.4–23.7	1.77–3.10	23.6–25.1	−12.6–−2.38
		GM(S)–6% Sand-bentonite	19.8–21.1	2.43–2.80	16.6–19.2	2.30–3.57
Mariappan et al. [33]	100, 200, 300	GM(S)–Native soil	15.6	0.00	-	-
		GM(T)–Native soil	23	0.00	-	-
Mariappan et al. [23]	100, 200, 300	GM(S)–Silt: bentonite (100:10)	5.2	0.00	-	-
		GM(S)–Sand: bentonite (100:10)	6.1	0.00	-	-
		GM(S)–Native soil	19.8	0.00	-	-
		GM(T)–Silt: bentonite (100:10)	9.1	0.00	-	-
		GM(T)–Sand: bentonite (100:10)	10.9	0.00	-	-
		GM(T)–Native soil	15.2	9.30	-	-
Frost et al. [22]	100, 300	GM(S)–Ottawa 20/30 sand	24.4–25.5	-	15.5–16.5	-
		GM(T)–Ottawa 20/30 sand	37.5–40.2	-	22.8–27.0	-
		GM(S)–Blasting sand	24.9–25.5	-	19.5–20.0	-
		GM(T)–Blasting sand	37.1–37.2	-	26.2–27.2	-
Stark and Santoyo [32]	17, 50, 100, 200, 400	GM(S)–Urbana glacial till	13–14	-	-	-
		GM(T)–Urbana glacial till	30–36	-	-	-
		GM(S)–Ottawa sand GM(T)–Ottawa sand	19–22 27–32	- -	- -	- -

**Table 5:** Comparison of previous GM(S/T)–GT interface peak shear strength parameters with the results of present study.

Source	Shear Apparatus	Normal Stress (kPa)	GM(S)–GT Interface		GM(T)–GT Interface	
			Friction Angle δ (°)	Adhesion c (kPa)	Friction Angle δ (°)	Adhesion c (kPa)
Mitchell et al. [11]	A modified Karol–Warner direct shear testing apparatus	158, 316, 479	6–11	-	-	-
Stark et al. [6]	A modified Bromhead ring shear apparatus	48, 96, 192, 285	-	-	32	-
Triplett and Fox [12]	Pullout shear machine	1–486	9.9	0.3	31.7	7.4
Wasti and Özdüzgün [5]	Inclined board apparatus	5–50	12.28	3.34	27	30
Akpinar and Benson [34]	A double-interface shear device	7.5–49.5	11.6–14.5	-	25.4–27.7	-
Li and Gilbert [18]	A small-scale direct shear apparatus	-	-	-	24–28	-
Feng et al. [29]	A large direct shear test device	50, 100, 200	-	-	22.8	5.62
Present study	A large-scale composite apparatus	50, 100, 150, 200	12.96	-3.38	20.88	5.62
